# Quinine in Typhus and Typhoid Fevers

**Published:** 1852-07

**Authors:** 


*Quinine in Typhus and Typhoid Fevers.*—On this subject, Professor Bennett read a paper to the Medico-Chirurgical Society, on Wednesday evening. It appeared, that about the month of November last, Professors Bennett and Christison had received communications from Dr. Dundas, of Liverpool, mentioning the beneficial results he had found to follow the exhibition of quinine in the continued fevers of warm climates. So strong were the representations of Dr. Dundas, that Professor Bennett, whose period of clinical duty in the Infirmary had at that time just commenced, determined to give the remedy a fair trial. The doses Dr. Dundas recommended were ten grains every second hour. Dr. Bennett had given quinine in seven out of fourteen cases of typhus or typhoid fever admitted into the Royal Infirmary during the period from November to the end of February. In all seven the physiological effects of the remedy had been produced. In most of the cases, the seventh was the day of the disease on which the treatment of quinine was begun, in some it was later; one patient, however, had it on the sixth day, and none later than the tenth. In one case, 205 grains of quinine had been taken in eighteen doses. In five others about 80 grains had been taken. Dr. Bennett had in none of these instances found the quinine to cut short the disease, or in any way favorably to influence its progress. In one case convalescence had been delayed till the forty-second day. At the same time, Dr. Bennett thought it proper, in bringing the subject before the Society, to mention, that the experience of some other physicians had been more favorable. Dr. Graves, of Dublin, for example, had written to Dr. Dundas to that effect, and so had Dr. Kelly, of Drogheda, who had treated eight cases with the happiest results. Professor Christison's experience had as yet been limited to one case, that of a girl who had contracted typhoid fever in the hospital when recovering from another disease. In it the employment of the remedy had certainly produced no favorable effect—it was now the forty-second day, and no marked convalescence had commenced. He regarded Dr. Bennett's experiments as of additional value from having been made *coram publico*, in the presence of a large number of intelligent students, many of whom had been from time to time examined on the very cases themselves. Important as the inquiry was, he much feared that the discovery of quinine as an antidote in continued fever would prove of but little avail, unless the additional discovery of new cinchona forests was also made.

Dr. William Robertson had also administered the quinine in some cases, in none with benefit. He had in two cases observed very alarming symptoms to follow its exhibition, a state of almost complete coma having been induced.—*Medical Times.*

